# Identification of novel genes whose expression in adipose tissue affects body fat mass and distribution: an RNA-Seq and Mendelian Randomization study

**DOI:** 10.1038/s41431-022-01161-3

**Published:** 2022-08-11

**Authors:** Stefan Konigorski, Jürgen Janke, Giannino Patone, Manuela M. Bergmann, Christoph Lippert, Norbert Hübner, Rudolf Kaaks, Heiner Boeing, Tobias Pischon

**Affiliations:** 1 Digital Health & Machine Learning Research Group, Hasso Plattner Institute for Digital Engineering, University of Potsdam, Potsdam, Germany; 2 Molecular Epidemiology Research Group, Max Delbrück Center (MDC) for Molecular Medicine in the Helmholtz Association, Berlin, Germany; 3 Hasso Plattner Institute for Digital Health at Mount Sinai, Icahn School of Medicine at Mount Sinai, New York, NY USA; 4 Genetics and Genomics of Cardiovascular Diseases Research Group, Max Delbrück Center (MDC) for Molecular Medicine in the Helmholtz Association, Berlin, Germany; 5 German Institute of Human Nutrition Potsdam–Rehbrücke (DIfE), Nuthetal, Germany; 6 DZHK (German Center for Cardiovascular Research) partner site Berlin, Berlin, Germany; 7 Charité Universitätsmedizin Berlin, Berlin, Germany; 8 Department of Cancer Epidemiology, German Cancer Research Center (DKFZ), Heidelberg, Germany; 9 MDC Biobank, Max Delbrück Center for Molecular Medicine (MDC) in the Helmholtz Association, Berlin, Germany; 10 BIH Biobank, Berlin Institute of Health, Berlin, Germany

**Keywords:** Gene expression, High-throughput screening, Genetic association study

## Abstract

Many studies have shown that abdominal adiposity is more strongly related to health risks than peripheral adiposity. However, the underlying pathways are still poorly understood. In this cross-sectional study using data from RNA-sequencing experiments and whole-body MRI scans of 200 participants in the EPIC-Potsdam cohort, our aim was to identify novel genes whose gene expression in subcutaneous adipose tissue has an effect on body fat mass (BFM) and body fat distribution (BFD). The analysis identified 625 genes associated with adiposity, of which 531 encode a known protein and 487 are novel candidate genes for obesity. Enrichment analyses indicated that BFM-associated genes were characterized by their higher than expected involvement in cellular, regulatory and immune system processes, and BFD-associated genes by their involvement in cellular, metabolic, and regulatory processes. Mendelian Randomization analyses suggested that the gene expression of 69 genes was causally related to BFM and BFD. Six genes were replicated in UK Biobank. In this study, we identified novel genes for BFM and BFD that are BFM- and BFD-specific, involved in different molecular processes, and whose up-/downregulated gene expression may causally contribute to obesity.

## Introduction

Obesity is an established risk factor for many chronic diseases and for premature death. Numerous studies have shown that abdominal adiposity is more strongly related to health risks than peripheral adiposity [1]. In line with this observation is evidence that visceral adipose tissue (VAT, which is the major compartment that determines abdominal adiposity) is metabolically more active than subcutaneous adipose tissue (SAT, which is the major determinant of peripheral adiposity). SAT might even have protective effects [2].

Previous authors estimated a heritability between 40% and 90% for obesity, expressed as body mass index (BMI) or absolute fat mass [3, 4], and slightly lower heritability between 15% and 60% for body fat distribution, expressed as waist to hip ratio (WHR) or various other ratios of fat mass in different body compartments [5–7]. These estimates have partly come out of large genetic association studies with obesity traits, which have identified many associated genetic loci, again with a higher number of identified loci associated with fat mass compared to fat distribution [7–10]. However, for many of those loci and genes, it is unclear how they affect obesity and particularly body fat distribution. In addition, their functional attributes are poorly understood. There are a few studies in humans that have investigated the association of genes with obesity on the transcriptomic or proteomic level [11–20]. Campbell et al. [11], Armenise et al. [15], Day et al. [16] and Kerr et al. [17] investigated the association between weight loss and gene expression in adipose tissue as well as in whole blood. The most often implicated pathways were inflammatory pathways [12, 17–20] and lipid as well as glucose pathways [12, 17, 19], with evidence that they are upregulated and changed in obesity. These changes might in turn be part of the role of obesity in cancer [11, 12]. Other studies based on RNA-sequencing of subcutaneous adipose tissue focused on identifying eQTLs which was followed-up by colocalization analyses with GWAS hits for cardiometabolic traits [14] or investigated cellular heterogeneity of gene expression in adipose tissue [13]. Little is known about whether gene expression in SAT affects body fat distribution, and if different genes are implicated in body fat mass and body fat distribution. Such knowledge may help to gain information about biological processes contributing to adiposity and body fat distribution as major determinants of health risks.

In this study, our aim was therefore to identify and to characterize novel genes whose gene expression in SAT is associated with obesity traits of body fat mass and body fat distribution. We performed cross-sectional analyses of ribonucleic acid (RNA)-sequencing experiments from abdominal SAT biopsies and whole-body magnetic resonance imaging (MRI) scans on 200 participants in the EPIC Potsdam study. SAT mass and the ratio of SAT and total adipose tissue (TAT) were obtained from whole-body MRI scans as measures of fat mass and fat distribution. In the analysis, we first investigated the association of gene expression with SAT and SAT/TAT. For the association tests, we used a recently developed method based on joint copula models [21] to improve power of association tests with multiple phenotypes. We followed-up the results with a gene ontology term enrichment analysis which indicated that SAT-associated genes were characterized by their higher than expected involvement in cellular, regulatory and immune system processes, and SAT/TAT-associated genes by their involvement in cellular, metabolic, and regulatory processes. Mendelian Randomization (MR) analyses confirmed that these novel genes are specific for body fat mass or body fat distribution, i.e. implicating different molecular processes, and suggested that the up-regulation or downregulation of the gene expression may causally contribute to obesity. Finally, we replicated the results using UK Biobank data, where we imputed AT gene expression based on exome sequencing data and weights learned in the analysis of the EPIC Potsdam study.

## Methods

### Study population

This study was conducted in a sub–cohort of EPIC Potsdam within the large European Prospective Investigation into Cancer and Nutrition (EPIC) study [22]. EPIC Potsdam is an ongoing cohort study among 27,548 persons aged 35–65 at recruitment between 1994–1998 from the general population of the city of Potsdam and surrounding area in Germany [22]. From 2010 to 2013, a random sample of 1472 participants was re–invited to the study center of whom 816 agreed to participate [23, 24].

MRI scans were obtained to assess body compartments from 594 participants on a separate visit [25]. Based on automated segmentation algorithms of the MRI scans [26, 27], for the analysis in this manuscript, SAT mass (fat mass in subcutaneous adipose tissue) was extracted as a measure of absolute fat mass, and the ratio of SAT and total adipose tissue (TAT) mass, SAT/TAT, as a measure of body fat distribution [28].

Subcutaneous adipose tissue biopsies were taken from 278 participants with sufficient material extracted from 200 participants [28]. The total RNA was extracted for RNA-sequencing (RNA-Seq). Single nucleotide variants (SNVs) were called from the RNA-Seq data for the 200 participants. For 160 of the participants, MRI measurements were available, which therefore constituted the sample for this study. In comparison to the full EPIC–Potsdam cohort as well as to the 816 participants of the substudy, these 160 probands were very similar regarding their age and sex distribution, disease prevalence, and anthropometric measures (data not shown). Sex was set to equal the assessed gender.

For a replication analysis of the associated genes, we used UK Biobank data (www.ukbiobank.ac.uk). The UK Biobank is a prospective cohort study encompassing data of about 500,000 participants (40–69 years of age at baseline) from Great Britain [29], including whole-exome sequencing data of about 49,960 participants at the time of analysis [30] and MRI scans of about 10,000 participants [31]. In the replication, we analyzed the subset of unrelated (i.e. excluding one person of each pair with greater than 3rd-degree relatedness) white British participants (based on self-report and their genetic principal components), which yielded a sample size of *n* = 4904.

### Assessment of gene expression in subcutaneous adipose tissue

The multiplexed probes were sequenced on the Illumina HiSeq 2000 platform. After the sequencing, the reads were aligned to hg38 (GRCh38.78) using TopHat2 version 2.0.12 [32] and Bowtie 2 version 2.0.6.0 [33] and quality-controlled. In order to obtain gene expression measures, the aligned reads were counted using htseq-count [34] and trimmed mean of M values (TMM)-normalized transcripts per million (TPM) counts were obtained. [35, 36] Finally, the normalized read counts were quality-controlled and low-expressed genes (expressed in less than 25% or the participants) were filtered, which yielded 30,917 genes for the main analysis.

### Assessment of genetic variation

In order to investigate genetic variants, SNVs (in coding regions) were called from the RNA-Seq reads using the mpileup tool of bcftools version 1.9 [37] and further quality-controlled, trimmed and imputed. For the complete-case analysis in the sample of *n* = 160, 4,776,233 autosomal biallelic non-monomorphic quality-controlled SNVs were available.

See Supplementary Text for more details regarding the study population, pre-processing of the RNA-seq data, quality control steps, and details on genetic variation processing.

### Statistical analysis

All analyses were performed in R 3.6.3 [38]. SAT mass was log-transformed for all analyses to yield a normally-distributed measure. The Yeo-Johnson transformation [39] was used to remove skewness and yield normally-distributed gene expression measures (based on the TMM-normalized TPM counts) for all analyses described in the following. SAT/TAT was not transformed.

In the first part of the analysis, the SAT gene expression of each of the 30,917 genes was tested for its association with SAT and SAT/TAT separately, in copula models [21, 40, 41] of the joint distribution of SAT and SAT/TAT conditional on the respective gene expression and the covariates age, sex, smoking status, physical activity and education. Copula functions can be used as a flexible tool to model the joint distribution of multiple outcomes, here SAT and SAT/TAT. By modeling the dependence of SAT and SAT/TAT, which had dependence Kendall’s *τ* = 0.36, the power of the association tests can be increased. In more detail, the joint distribution *F* of SAT and SAT/TAT was constructed using the copula function *Cψ*, $F\left( {{{{{{{{\mathrm{SAT}}}}}}}},{{{{{{{\mathrm{SAT}}}}}}}}/{{{{{{{\mathrm{TAT|}}}}}}}}{{{{{{{\boldsymbol{x}}}}}}}}} \right) = C_\psi \left( {F_1\left( {{{{{{{{\mathrm{SAT|}}}}}}}}{{{{{{{\boldsymbol{x}}}}}}}}} \right),F_2\left( {{{{{{{{\mathrm{SAT}}}}}}}}/{{{{{{{\mathrm{TAT|}}}}}}}}{{{{{{{\boldsymbol{x}}}}}}}}} \right)} \right)$, with marginal models

1 $${{{{{{{\mathrm{SAT}}}}}}}} = \gamma _0 + \gamma _1x_1 + \gamma _2x_2 + \gamma _3x_3 + \gamma _4x_4 + \gamma _5x_5 + \beta _jg_j + \varepsilon$$

2 $${{{{{{{\mathrm{SAT}}}}}}}}/{{{{{{{\mathrm{TAT}}}}}}}} = \gamma _0^\prime + \gamma _1^\prime x_1 + \gamma _2^\prime x_2 + \gamma _3^\prime x_3 + \gamma _4^\prime x_4 + \gamma _5^\prime x_5 + \beta _j^\prime g_j + \varepsilon ^\prime$$

and the 2-parameter copula function $C_{\it{\uppsi }}( {u_1,u_2, \ldots u_p,\varphi ,\theta } ) = \{[ \mathop {\sum}\nolimits_{l = 1}^p {( {u_l^{ - \varphi }} - 1)^\theta}] ^{1/\theta} + 1\}^{ - 1/\varphi }$ with 0 ≤ *u*_1_,*u*_2_ ≤ 1 and ${\it{\uppsi }} = ( {\varphi ,\theta } )^T,\varphi \, > \, 0,\theta \ge 1.$ Here, *F*_1_ and *F*_2_ are the marginal distributions of SAT and SAT/TAT and ${{{{{{{\boldsymbol{x}}}}}}}} = (x_1,x_2,x_3,x_4,x_5,g_j)^T$ includes the gene expression *g*_*j*_ and covariates $x_1 \ldots x_5$ sex, age, smoking, physical activity, education. Hence, in the marginal models, parameter estimates of *β*_*j*_ and $\beta _j^\prime$ can be interpreted analogously to linear regression models and quantify the change in SAT or SAT/TAT for a 1 unit increase in gene expression, given the covariates. The copula models were fitted sequentially for the gene expression of each gene *g*_*j*_, $j = 1, \ldots ,30,917$, and the large-sample Wald test statistics were computed to test the null hypotheses $H_{0j}:\beta _j = 0$ (vs. $H_{Aj}:\beta _j \,\ne\, 0$) and $H_{0j}:\beta _j^\prime = 0$ (vs. $H_{Aj}:\beta _j^\prime \,\ne\, 0$) using the cjamp function in the CJAMP (copula-based joint analysis of multiple phenotypes) R package [42].

Next, the obesity-associated genes identified in the above analyses were characterized regarding their functional properties, performing a gene ontology (GO)-term enrichment analysis in order to identify which gene ontology terms are enriched (under-/overrepresented) in the obesity-associated genes compared to all 30,917 analyzed genes. For details see the Supplementary Text.

In the subsequent analyses, the focus was restricted to the autosomal obesity-associated genes, and it was investigated how many of the associated genes have been found to be associated with obesity or body fat distribution in previous studies. For this, the NCBI gene database (accessed at https://www.ncbi.nlm.nih.gov/gene on July 25, 2020) was searched for genes associated with “obesity”, and all entries were extracted filtering for humans. Furthermore, the GWAS Catalog [10] (accessed at https://www.ebi.ac.uk/gwas/ on July 25, 2020) was searched for Experimental Factor Ontology (EFO) traits “obesity”, “fat body mass”, “body mass index”, “body composition measurement“, “body fat distribution”, “BMI-adjusted waist-hip ratio”, “visceral:subcutaneous adipose tissue ratio” and “visceral:total adipose tissue ratio”, and all SNVs (associations) with a *p* value <10^−5^ for all relevant reported traits and child traits were extracted by restricting the results to main overall effects (i.e. ignoring interaction effects, subgroup analyses, and proxy traits such as protein levels of obesity as traits), and restricting to body fat distribution traits of the trunk/abdomen (i.e. ignoring e.g. leg fat distribution). Finally, we queried the AstraZeneca PheWAS Portal (accessed at https://azphewas.com on June 25, 2022) which is based on a recent phenome-wide association study [43] of 18,762 genes and 2,108,983 SNVs using exome-sequencing data in UK Biobank. We extracted genes that were associated (i.e. had a *p* value <0.05/18,762 in any of the 11 performed collapsing gene-level tests, or contained a SNV with *p* value <0.05/2,108,983 in genotypic variant-level association tests) with BMI, waist circumference, whole body fat mass, abdominal SAT mass or VAT mass. For all lists, gene symbols were extracted and in order to match this list with the list of the genes associated with SAT and SAT/TAT in our study, gene symbols were extracted from the Ensembl identifiers (ID) using the biological DataBase network (accessed at https://biodbnet-abcc.ncifcrf.gov/db/db2db.php on July 25, 2020). Next, we investigated for each of the identified genes whether they encode a known protein, also by using the biological DataBase network––in more detail, by inputting the Ensembl ID of the genes and outputting the encoded UniProt protein name.

Next, we investigated the causal role of the identified genes in obesity. To this aim, we performed a Mendelian randomization (MR) study to investigate the association of genetically-determined gene expression with SAT mass and SAT/TAT. For this, all SNVs were included in the MR analysis that (i) have been identified as single-tissue cis expression quantitative trait loci (eQTLs) for SAT gene expression in Genotype-Tissue Expression (GTEx) version 8 for that respective gene (obtained from https://www.gtexportal.org/home/datasets), (ii) were not associated with any confounder of the gene expression–SAT, gene expression–SAT/TAT association, i.e. not associated with covariates sex, age, smoking, physical activity, education (tested using Wald tests of the regression coefficients in linear regression models or Fisher’s exact tests, with statistical significance threshold 0.001), (iii) were not associated with SAT and SAT/TAT, respectively, conditional on the expression of the respective gene and confounders sex, age, smoking, physical activity, education (tested in Wald tests of the regression coefficients in linear regression models, with statistical significance threshold 0.001), and (iv) with further filtering by excluding one SNV of each SNV pair with Spearman correlation greater than 0.9 (or smaller than −0.9). The analysis was performed using the *mr_ivw* function in the Mendelian Randomization R package [44], with the “weights =’delta’” option, psi being set to the sample correlation between gene expression and obesity measure, otherwise default settings and using the “correl=TRUE” option, which computes the inverse-variance weighted method (IVW) and allows to incorporate multiple correlated SNVs.

For a replication of the causally associated genes, we used UK Biobank data. Based on the filtered SNVs that were used in the Mendelian Randomization analysis, and their weights from a multiple linear regression model predicting the respective gene expression of the gene in the EPIC-Potsdam data, we imputed SAT gene expression based on the whole-exome sequencing data in the UK Biobank data. Then, we tested the association of this imputed genetically-determined SAT gene expression with abdominal SAT (aSAT) mass and aSAT/(aSAT+VAT) from MRI scans, in a linear regression model adjusting for age, sex, smoking and education.

## Results

### Description of the participants’ characteristics

The characteristics of the study population from the EPIC Potsdam study are shown in Table 1. There were slightly more women than men, and participants constituted an older and predominantly healthy sample from the general population.

**Table 1:** Sex-stratified characteristics of the study population from the EPIC-Potsdam substudy (*n*=160).

Characteristics	Women	Men
Sample size	88	72
Age, years	62.7 (8.4)	66.9 (8.3)
Smoking, %		
Never	54.5	29.2
Former	31.8	55.6
Current	13.6	15.3
CPAI, %		
Inactive	8.0	8.3
Moderately inactive	26.1	29.1
Moderately active	35.2	33.3
Active	30.7	29.2
Vocational training, %		
No vocational training/ vocational training	40.9	34.7
Technical college	26.1	11.1
University	31.8	54.2
Myocardial infarction, %	0	1.4
Stroke, %	0	0
Heart failure, %	0	0
Diabetes, %	2.3	11.1
BMI, kg/m^2^	27.8 (4.3)	28.1 (3.9)
SAT, kg*	20.1 (5.2)	14.8 (4.3)
TAT, kg*	23.7 (5.4)	21.0 (5.9)

Values are relative frequencies, mean and SD, or *median and median absolute deviation.

*CPAI* Cambridge physical activity index, *BMI* body mass index, *AT* adipose tissue, *SAT* subcutaneous AT, *TAT* total AT.

### Screen of associations between gene expression and obesity

In the first part of the analysis, the SAT gene expression of each of the quality-controlled 30,917 genes was tested for its association with SAT and SAT/TAT separately. The transcriptome-wide association analysis using C-JAMP identified 441 genes associated with SAT mass and 225 associated with SAT/TAT after a respective Bonferroni-correction for multiple testing of the 30,917 genes, i.e. with a respective *p* value cutoff of 0.05/30,917. Of these genes, 41 overlapped so that in total, 625 genes were identified to be associated with adiposity (see Table 2). For sensitivity checks, standard univariate regression models were computed of the respective marginal models. The results showed that there was a large overlap of the associated genes identified in the copula analysis and the regression analysis, also with similar ranking (see Figure S1). The copula-based analysis identified more associated genes as compared to standard linear regression of the marginal models (410 with respect to SAT and 121 genes with respect to SAT/TAT).

**Table 2:** Overview of the number of genes associated with only SAT, only SAT/TAT, with both SAT and SAT/TAT, and their total sum, in the analysis of 30,917 genes in the study population from the EPIC-Potsdam substudy (*n*=160).

Description	SAT only	SAT/TAT only	SAT and SAT/TAT	Total
Associated genes^a^	400	184	41	625
Associated autosomal genes^a^	392	177	38	607
Associated autosomal genes that are known^b^	69	38	13	120
Associated autosomal genes that are novel^b^	323	139	25	487
Associated autosomal genes which encode known protein^c^	338	158	35	531

^a^after Bonferroni-correction for multiple testing of the 30,917 genes.

^b^Overlap with genes that have been associated with obesity and body fat distribution in previous studies on the molecular or genetic level. These lists have extracted on July 25, 2020 from the (i) NCBI gene database (https://www.ncbi.nlm.nih.gov/gene) by searching and extracting all associated with “obesity” in humans, and (ii) GWAS Catalog (https://www.ebi.ac.uk/gwas/), where all SNVs (associations) with a *p* value <10^−5^ for all relevant reported traits and child traits were extracted when searching for EFO (Experimental Factor Ontology) traits “obesity”, “fat body mass”, “body mass index”, “body composition measurement“, “body fat distribution”, “BMI-adjusted waist-hip ratio”, “visceral:subcutaneous adipose tissue ratio” and “visceral:total adipose tissue ratio”, and restricting the results to main overall effects (i.e. ignoring interaction effects, subgroup analyses, and proxy traits such as protein levels of obesity as traits), and restricting to body fat distribution traits of the trunk/abdomen (i.e. ignoring e.g. leg fat distribution). (iii) Further, the AstraZeneca PheWAS Portal was accessed at https://azphewas.com on June 25, 2022 and genes were extracted that were associated with BMI, waist circumference, whole body fat mass, abdominal SAT mass or VAT mass in gene-level or variant level association tests. (i) yielded 1962 genes, (ii) SNVs in 2509 genes, and (iii) 23 genes and SNPs in 562 genes, which yields in total 4460 known candidate genes for obesity and body composition.

^c^Whether each gene encodes a known protein was checked using the UniProt annotation from https://biodbnet-abcc.ncifcrf.gov/db/db2db.php, queried on July 25, 2020.

### Characterization of the identified adiposity-associated genes

Of the 625 identified adiposity-associated genes (400 for SAT only, 184 for SAT/TAT only, 41 for both), 607 are autosomal genes and 18 are sex-chromosomal genes. Of the 607 autosomal genes, only 38 were associated with both SAT mass as well as SAT/TAT. In all further analyses described below, we focused on the 607 autosomal genes. In order to identify known and novel genes, the NCBI gene database, GWAS Catalog and AstraZeneca PheWAS Portal were searched which yielded 1962 genes, SNPs in 2509 genes, and as well as 23 genes and SNPs in 562 genes, respectively, for in total 4460 known genes associated with obesity and body composition. Of the 607 obesity-associated genes in our study, 120 have been found to be associated with adiposity in previous studies, such as the *LEP* gene encoding the adipokine leptin and several cytokines of the interleukin and tumor-necrosis-factor alpha families [45, 46]. Regarding a first functional characterization of the 607 genes, 531 encode a known protein. An overview of these numbers is given in Table 2.

Gene ontology (GO) term enrichment analyses indicated that the identified adiposity-associated genes are overrepresented in metabolic, cellular, regulatory and immune system processes, and that there are differences between those genes associated with body fat mass and those associated with body fat distribution. In more detail, there were 15 GO terms that were overrepresented in the 441 genes associated with SAT compared to the full pool of 30,917 genes, and 36 GO terms that were overrepresented in the 225 genes associated with SAT/TAT compared to the full pool of 30,917 genes. While the genes associated with body fat mass are mainly overrepresented in cellular, regulatory and immune system processes, those genes associated with body fat distribution are mainly overrepresented with cellular, metabolic, and regulatory processes (see Table 3 and Tables S1–S4). For example, there were 35 GO terms related to metabolic processes overrepresented in the genes associated with SAT/TAT, but no GO term related to metabolic processes overrepresented or underrepresented in the genes associated with SAT.

**Table 3:** Summary of the results of the GO term enrichment analysis in the study population from the EPIC-Potsdam substudy (*n*=160).

GO terms	SAT	SAT/TAT
Metabolic process	0	35
Cellular process	7	27
Immune system process	4	1
Localization	7	1
Locomotion	0	1
Response to stimulus	2	0
Biological regulation	1	0
Cell proliferation	1	0

There were 15 Gene Ontology (GO) terms that were (statistically significantly after multiple testing correction for all 6287 analyzed GO terms) overrepresented in the 441 genes associated with SAT compared to the full pool of 30,917 genes, and 36 GO terms that were (statistically significantly after multiple testing correction for all 6287 analyzed GO terms) overrepresented in the 225 genes associated with SAT/TAT compared to the full pool of 30,917 genes, using Fisher’s exact test. In this summary table, the number of highest-level parent GO terms of these overrepresented 15 (left panel) and 36 (right panel) GO terms is shown. For the full results of all 15 and 36 GO terms, see Tables S3, S4.

### Causal gene expression effects on obesity

Next, we investigated the causal role of the identified genes in obesity in more detail. To this aim, we performed a MR study to investigate the association of genetically-determined gene expression with SAT mass and SAT/TAT. The stringent filtering steps as described in the Methods section allowed to perform a MR analysis of 261 (of the 430) genes for SAT and of 122 (of the 215) genes for SAT/TAT, which each contained at least one single nucleotide variant (SNV) after the filtering steps. In the analysis, on average 12 and 9 SNVs were included per gene for SAT mass and SAT/TAT, respectively, (min=1, max=97 for SAT and min=1, max=38 for SAT/TAT). They explained on average 10% variance of the respective gene expression for SAT and 7% variance of the respective gene expression for SAT/TAT.

In the MR analyses, the genetically-determined gene expression of 53 genes was associated with SAT mass and of 16 genes with SAT/TAT, supporting a causal effect of gene expression on adiposity for these genes. Both sets of genes were non-overlapping. They explained on average 20% variance of the respective gene expression for SAT and 15% variance of the respective gene expression for SAT/TAT. Of these 69 genes, 57 are novel genes for obesity (i.e. have not been reported to be associated with adiposity in the NCBI database and GWAS Catalog), and 46 are novel and encode a known protein. An overview of these numbers is given in Table 4 and an overview of the p-values and results for all genes in Tables S5, S6.

**Table 4:** Overview of the number of genes associated with only SAT, only SAT/TAT, with both SAT and SAT/TAT, and their total sum, in Mendelian Randomization and replication analyses, of the 607 autosomal genes associated with SAT and/or with SAT/TAT (see Table 2) in the study population from the EPIC-Potsdam substudy (*n* = 160).

Description	SAT only	SAT/TAT only	SAT and SAT/TAT	Total
Associated autosomal genes^a^	392	177	38	607
Associated autosomal genes that were investigated in MR analysis^b^	234	95	27	356
Associated autosomal genes identified to be causal^c^	53	16	0	69
Associated autosomal genes identified to be causal and novel^c,d^	46	11	0	57
Associated autosomal genes identified to be causal, novel, and to encode a known protein^c,d^^,e^	36	10	0	46
Associated autosomal genes identified to be causal that were investigated in the UK Biobank replication analysis	38	10	0	48
Associated autosomal genes identified to be causal that were confirmed in the UK Biobank replication analysis	5	1	0	6

*AT* adipose tissue, *MR* Mendelian Randomization, *SAT* subcutaneous AT, *TAT* total AT.

^a^after Bonferroni-correction for multiple testing of the 30,917 genes.

^b^i.e. number of genes for which valid instruments were available to perform a MR analysis.

^c^To test whether the identified associations are causal, MR analysis were performed predicting SAT and SAT/TAT by genetically determined gene expression.

^d^Overlap with genes that have been associated with obesity and body fat distribution in previous studies on the molecular or genetic level. These lists have extracted on July 25, 2020 from the (i) NCBI gene database (https://www.ncbi.nlm.nih.gov/gene) by searching and extracting all associated with “obesity” in humans, and (ii) GWAS Catalog (https://www.ebi.ac.uk/gwas/), where all single nucleotide variant (SNV) associations with a *p* value <10^−5^ for all relevant reported traits and child traits were extracted when searching for EFO (Experimental Factor Ontology) traits “obesity”, “fat body mass”, “body mass index”, “body composition measurement“, “body fat distribution”, “BMI-adjusted waist-hip ratio”, “visceral:subcutaneous adipose tissue ratio” and “visceral:total adipose tissue ratio”, and restricting the results to main overall effects (i.e. ignoring interaction effects, subgroup analyses, and proxy traits such as protein levels of obesity as traits), and restricting to body fat distribution traits of the trunk/abdomen (i.e. ignoring e.g. leg fat distribution). (iii) Further, the AstraZeneca PheWAS Portal was accessed at https://azphewas.com on June 25, 2022 and genes were extracted that were associated with BMI, waist circumference, whole body fat mass, abdominal SAT mass or VAT mass in gene-level or variant level association tests. (i) yielded 1962 genes, (ii) SNVs in 2509 genes, and (iii) 23 genes and SNPs in 562 genes, which yields in total 4460 known candidate genes for obesity and body composition.

^e^Whether each gene encodes a known protein was checked using the UniProt annotation from https://biodbnet-abcc.ncifcrf.gov/db/db2db.php, queried on July 25, 2020.

### Replication of causally associated genes in UK Biobank

Finally, we used data generated from the participants of the UK Biobank for replication of the results. See Table S7 for characteristics of the study population containing *n* = 4904 participants. We were able to investigate 38 of the 53 genes for SAT mass and 10 of the 16 genes for SAT/TAT (see Table 4), with at least one SNV being available in the quality-filtered whole-exome sequencing data in the UK Biobank. For these genes, the SNVs explained on average 7% variance for SAT mass and 4% variance for SAT/TAT. Using the weights from the analysis of the EPIC-Potsdam dataset, the computed genetically-determined SAT gene expression score in UK Biobank of 5 genes was associated with SAT mass and of 1 gene was associated with aSAT/(aSAT+VAT). These 6 genes are *DBNDD1*, *PTPRU*, *ERAP1*, *ANKDD1A* and *LINC02798* for SAT and *MCC1* for aSAT/(aSAT+VAT), see Table 5 for an overview of these final genes with their functional annotation.

**Table 5:** Results and annotations for the 5 autosomal genes associated with SAT mass and 1 autosomal gene associated with SAT/TAT in the Mendelian Randomization analysis that were replicated in the UK Biobank analysis.

Gene information								Gene expression analysis			Mendelian Randomization analysis			Replication analysis		
Trait	Ensembl	Gene	Chr	Start	End	Novel	Protein	beta	SE	*p* value	no_var	R^2^	*p* value	no_var	R^2^	*p* value
SAT	ENSG00000003249	*DBNDD1*	16	90004865	90020128	YES	Dysbindin domain-containing protein 1	0,30	0,06	2,79 × 10^−7^	333	0,87	5,31 × 10^−4^	11	0,151	3,13 × 10^−3^
SAT	ENSG00000060656	*PTPRU*	1	29236516	29326813	YES	Receptor-type tyrosine-protein phosphatase U	0,34	0,06	4,97 × 10^−8^	39	0,03	5,05 × 10^−3^	1	0,012	2,63 × 10^−4^
SAT	ENSG00000164307	*ERAP1*	5	96760810	96808100	NO	Endoplasmic reticulum aminopeptidase 1	0,36	0,07	8,37 × 10^−7^	368	0,88	6,26 × 10^−11^	15	0,349	3,70 × 10^−2^
SAT	ENSG00000166839	*ANKDD1A*	15	64911902	64958700	YES	Ankyrin repeat and death domain-containing protein 1 A	0,37	0,06	8,39 × 10^−11^	128	0,29	1,09 × 10^−2^	6	0,077	2,37 × 10^−2^
SAT	ENSG00000227082	*LINC02798*	1	121396754	121463129	YES	--	0,32	0,06	5,82 × 10^−7^	250	0,57	4,38 × 10^−6^	1	0,001	2,63 × 10^−4^
SAT/TAT	ENSG00000078070	*MCCC1*	3	183015218	183116075	YES	Methylcrotonoyl-CoA carboxylase subunit alpha, mitochondrial	0,27	0,05	1,02 × 10^−7^	164	0,30	2,85 × 10^−2^	2	0,037	4,16 × 10^−2^

Shown are the Ensembl ID of the gene (“Ensembl”), the gene symbol (“Gene”), the genomic position in form of the chromosome number (“Chr”) as well as the start (“Start”) and end (“End”) position in base pairs of the gene; whether the gene is known to be associated with obesity (based on the NCBI gene,GWAS Catalog and AstraZeneca PheWAS Portal databases), the corresponding protein encoded by the gene if it is known (from UniProt); and results of the statistical analyses: the estimates of the effect size (“beta”; estimate of beta_j in the marginal model of C-JAMP in Eq. (1), or (2) respectively, in the main text) its standard error estimates (“SE”) and *p* value (“*p* value”) from the C-JAMP association analysis of SAT and SAT/TAT conditional on the gene expression and covariates; the results from the Mendelian randomization (MR) analysis in form of the number of SNVs in the gene that were incorporated in the MR analysis (“no_var”), the explained variance of the gene expression based on a multiple linear regression model containing these SNVs (“R^2^”), and the *p* value from the inverse-variance weighted MR method (“*p* value”); as well as the results of the replication analysis in form of the number of SNVs in the gene that were incorporated in the MR analysis (“no_var”), the explained variance of the gene expression based on a multiple linear regression model containing these SNVs (“R^2^”), and the *p* value from the linear regression analysis (“*p* value”).

The following gene ontology terms of the Biological Process (BP) ontology are associated with each of the genes, obtained using the annFUN.org() function in the topGO R package:

*DBNDD1*: --

*PTPRU*: protein dephosphorylation; cell adhesion; transmembrane receptor protein tyrosine phosphatase signaling pathway; negative regulation of cell proliferation; cell differentiation; negative regulation of cell migration; animal organ regeneration; homotypic cell-cell adhesion; protein localization to cell surface; peptidyl-tyrosine dephosphorylation; response to glucocorticoid; negative regulation of canonical Wnt signaling pathway; positive regulation of cell-cell adhesion mediated by cadherin

*ERAP1*: angiogenesis; adaptive immune response; antigen processing and presentation of peptide antigen via MHC class I; membrane protein ectodomain proteolysis; regulation of blood pressure; response to bacterium; antigen processing and presentation of endogenous peptide antigen via MHC class I; regulation of innate immune response; fat cell differentiation; positive regulation of angiogenesis

*ANKDD1A*: signal transduction

LINC02798: --

*MCCC1*: leucine catabolic process; biotin metabolic process; branched-chain amino acid catabolic process; protein heterooligomerization

## Discussion

In this study, we identified 487 novel candidate genes for adiposity and 120 genes that have previously been found to be related to adiposity. The MR analysis indicates that for 69 genes, there is evidence for a causal role of their gene expression in adiposity. Importantly, 57 of these 69 genes––46 genes for body fat mass and 11 genes for body fat distribution––have not been established as adiposity genes in previous studies, and are interesting novel candidate genes whose gene expression may causally affect adiposity. Six genes were confirmed in stringent replication analysis using UK Biobank data.

Investigating these genes in follow-up studies can provide novel evidence of the molecular correlates and pathways underlying both abdominal adiposity as well as peripheral adiposity, and provide a fine-grained view on the different obesity traits that altogether constitute an established risk factor for many chronic diseases and for premature death. Interestingly, the results of our study provide ample view that body fat mass and body fat distribution are distinct phenotypes with distinct molecular correlates and underlying pathways. This was observed in the results of the transcriptomic association analysis, where 441 genes were associated with SAT, 225 genes were associated with SAT/TAT, and only 41 of these genes were overlapping. All subsequent follow-up analyses including the MR analysis fortified this separation further and revealed non-overlapping sets of genes. In addition to these separate sets of associated genes, the GO-term enrichment analyses provided further evidence. Their results indicated that SAT-associated genes were characterized by their higher than expected involvement in cellular, regulatory and immune system processes, and SAT/TAT-associated genes by their involvement in cellular, metabolic, and regulatory processes.

We investigated the causal role of the identified genes in obesity regarding the question whether they affect obesity causally, e.g. through an upregulation or downregulation of their gene expression which contributes to a metabolic imbalance which in turn contributes to obesity. The results of the Mendelian Randomization analysis suggest a causal effect of gene expression of 53 genes on SAT mass and of 16 genes on SAT/TAT. The replication analyses of these results in the UK Biobank provide support for a causal role of the gene expression of six genes on adiposity: *DBNDD1*, *PTPRU*, *ERAP1*, *ANKDD1A* and *LINC02798* for body fat mass and *MCC1* for body fat distribution. All these genes have not been listed in neither the NCBI database nor the GWAS Catalog as being associated with adiposity and are novel candidate genes. Only the *ERAP1* gene has recently been associated in rare-variant association studies with BMI and body fat mass [43]. Further, all genes except for the Long Intergenic Non-Protein Coding RNA 2798 (*LINC02798*) have known proteins that could be further candidate biomarkers of interest. Regarding *DBNDD1* (Dysbindin Domain Containing 1), there is evidence of its involvement in gluco-metabolic pathways [47] and through its function of binding dystrobrevin, a protein involved in intracellular processes in muscle tissue, has also some evidence for an involvement in type 2 diabetes [48]. *PTPRU* (Protein Tyrosine Phosphatase Receptor Type U) encodes a protein of the protein tyrosine phosphatase (PTP) family, and is a key regulator of cell communication through regulating cellular phosphotyrosine levels. The PTP family is involved, among others, in different metabolic pathways [49] and regulatory processes of cancer and diabetes [50]. Another candidate of interest for follow-up investigations is *ERAP1*, which encodes the endoplasmic reticulum aminopeptidase 1 and is involved in MHC class I antigen processing, hence immune processes [51], as well as in peptide catabolic processes and type 1 diabetes [52]. *ANKDD1A* (Ankyrin Repeat And Death Domain Containing 1 A) is a functional tumor suppressor gene and involved in signal transduction [53]. Similarly to *MCC1* (Methylcrotonoyl-CoA Carboxylase 1), the involvement of *ANKDD1A* in metabolic and catabolic processes is still unclear. In addition to these genes and proteins, further interesting genes identified by the MR analyses but that could not be investigated in the UK Biobank replication are, for example, *CD44*, *PLCXD3*, *ANG*, *GPR39* and *GALNT10* [54].

Our study has some limitations. First, the analyses of the EPIC-Potsdam and UK Biobank cohorts were based on comparably small sample sizes, and the MR analyses as well as replication analysis were based on weak instruments. Nevertheless, we found a high number of genes to be associated with our outcomes in the gene expression analysis. This was made possible by high-quality phenotyping, analysis of quantitative phenotypes, deep RNA-sequencing data, extensive quality-control to reduce measurement error and imprecise measures, and the use of powerful statistical modeling using copula models. The association tests based on copula tests yielded more associations compared to linear regression models, and their validity to keep the nominal type I error is supported by detailed evaluations of the copula models and Wald tests in previous studies [21, 41]. These strengths of our study counterbalanced the smaller sample size compared to published studies on gene expression in subcutaneous adipose tissue that didn’t have MRI data available [14] or another focus in the analysis on cell-type composition [13]. In the choice between using more liberal instruments in the MR analysis and using more stringent filtering criteria on the SNVs, we opted for the latter to minimize risk of bias. As such, we believe that our results provide a lower bound on the genes whose gene expression in SAT is causally linked to adiposity. Similarly, only very few SNVs could be used in the replication analysis in the UK Biobank to impute SAT gene expression based on the whole-exome sequencing data. Still, the imputed gene expression of 6 genes was associated with body fat mass and body fat distribution, supporting the robustness of our analyses and results. As further limitation, the genotype calls in our study were not available from genotyping microarrays or DNA-sequencing and were called from the RNA-sequencing data. Due to again stringent filtering steps, few SNVs remained and were used in imputation. In our opinion, this might have rather decreased the power of the subsequent MR analysis, instead of increasing bias and false positive findings. As another point of discussion, about 10% of participants in our sample took antidiabetic drugs, which might affect gene expression of some genes. However, different drugs might have different effects and detailed information on drugs was not available. Therefore, we opted not to add antidiabetic medication use as a covariate in the copula model, which was supported by sensitivity checks we performed in an earlier study where results of association studies did not change [28]. Even if the association tests of some genes might have been affected by this, our choice of following up the identified genes in a Mendelian Randomization analysis ensured that these results would not be affected. Finally, in our study, we only investigated gene expression in subcutaneous adipose tissue. Without the assessment of VAT gene expression and secretion rates from SAT and VAT, however, parts of the overall molecular picture remain unclear. For example, in terms of molecular mechanisms, it cannot be ruled out that the gene expression in VAT is upregulated or downregulated in parallel to the gene expression in SAT. However, assessing VAT in a population–based study is rarely possible, and VAT gene expression measured after bariatric or other surgeries might not allow for a valid approximation of the metabolic activity in the general population.

In summary, we identified novel adiposity genes that are fat mass specific and fat distribution-specific, involved in different molecular processes, and whose upregulated or downregulated gene expression may causally contribute to obesity. These findings can provide guidance for future work in finding pieces in the puzzle of molecular mechanisms contributing to adiposity.

### Supplementary information

Supplementary Material

Supplementary Table 5

Supplementary Table 6

## Data Availability

For the analyses described in this manuscript, the file Adipose_Subcutaneous.v8.signif_variant_gene_pairs.txt.gz from GTEx_Analysis_v8_eQTL.tar was obtained from the GTEx Portal https://www.gtexportal.org/home/datasets on 05/18/2020. The UK Biobank data can be requested upon application at https://www.ukbiobank.ac.uk/register-apply/. The analyzed datasets from the still ongoing EPIC-Potsdam study have ethical and legal restrictions for public deposition due to the data protection rules applicable to its informed consent. The gene expression data and phenotypic data will therefore be available upon application with an appropriate research proposal which goes through a process of review and evaluation. To request the data, please contact Prof. Matthias Schulze at mschulze@dife.de. All bioinformatic processing and statistical analyses were conducted using publicly available software as referenced in the Methods section and Supplementary Text.
