# Correction to: Identification of novel genes whose expression in adipose tissue affects body fat mass and distribution: an RNA-Seq and Mendelian Randomization study

**DOI:** 10.1038/s41431-022-01183-x

**Published:** 2022-08-29

**Authors:** Stefan Konigorski, Jürgen Janke, Giannino Patone, Manuela M. Bergmann, Christoph Lippert, Norbert Hübner, Rudolf Kaaks, Heiner Boeing, Tobias Pischon

**Affiliations:** 1 Digital Health & Machine Learning Research Group, Hasso Plattner Institute for Digital Engineering, University of Potsdam, Potsdam, Germany; 2 Molecular Epidemiology Research Group, Max Delbrück Center (MDC) for Molecular Medicine in the Helmholtz Association, Berlin, Germany; 3 Hasso Plattner Institute for Digital Health at Mount Sinai, Icahn School of Medicine at Mount Sinai, New York, NY USA; 4 Genetics and Genomics of Cardiovascular Diseases Research Group, Max Delbrück Center (MDC) for Molecular Medicine in the Helmholtz Association, Berlin, Germany; 5 German Institute of Human Nutrition Potsdam–Rehbrücke (DIfE), Nuthetal, Germany; 6 DZHK (German Center for Cardiovascular Research) partner site Berlin, Berlin, Germany; 7 Charité Universitätsmedizin Berlin, Berlin, Germany; 8 Department of Cancer Epidemiology, German Cancer Research Center (DKFZ), Heidelberg, Germany; 9 MDC Biobank, Max Delbrück Center for Molecular Medicine (MDC) in the Helmholtz Association, Berlin, Germany; 10 BIH Biobank, Berlin Institute of Health, Berlin, Germany

**Keywords:** Gene expression, High-throughput screening, Genetic association study

Correction to: *European Journal of Human Genetics* 10.1038/s41431-022-01161-3, published online 11 August 2022

In this article the affiliation details for Tobias Pischon were incorrectly given as the affiliation 1 but should have been the affiliation 2.

