# Supplementary material for: Identification of novel genes whose expression in adipose tissue affects body fat mass and distribution: an RNA-Seq and Mendelian Randomization study
Source: Eur J Hum Genet. 2022 Aug 11;32(9):1127–35. doi: 10.1038/s41431-022-01161-3 (PMC11369295; doi:10.1038/s41431-022-01161-3)
Supplement: Supplementary file 1 — Supplementary Material [file 41431_2022_1161_MOESM1_ESM.pdf]

Identification of novel genes whose expression in adipose tissue affects body fat mass and distribution: An RNA-Seq and Mendelian Randomization study

SUPPLEMENTARY MATERIAL

Content

Supplementary Text 2

MRI scans 2

RNA extraction from SAT biopsies 2

Library preparation & multiplexing 3

Sequencing and demultiplexing 3

Quality control of the raw sequencing reads 4

Read alignment 4

Quality control of the aligned reads 5

Read counts as raw measures of gene expression levels 5

Quality control and normalization of read counts 5

Transformations of normalized read counts 7

Assessment of genetic variation 8

GO-term enrichment analysis 8

References 9

Supplementary Tables 11

Supplementary Figures 19

Supplementary Text: Details on study population, RNA sequencing data preprocessing & quality control steps

MRI scans

MRI scans were obtained to assess body compartments from 594 participants. The measurements were performed with a 1.5T MRT scanner ("Magnetom Avanto", Siemens, Erlangen, Germany) and the Vibe Dixon sequence. The Vibe-Dixon sequence is a special MRI protocol for body fat analysis which separates the fat from tissue water. Automated segmentation algorithms of the MRI scans were used to quantify the fat mass in different body compartments with high repeatability and reproducibility.

RNA extraction from SAT biopsies

Subcutaneous adipose tissue biopsies were taken from 278 participants using a needle aspiration method with sufficient material extracted from 200 participants. For details regarding the subcutaneous AT biopsies, see Konigorski et al. (2019)[1]. From the SAT biopsies, the total RNA, genomic DNA, and total protein from the fat tissue samples were purified and separated using the Qiagen All Prep DNA/RNA Mini Kit. The purified genomic DNA has an average length of 15-30 kb. Regarding the RNA, only RNA molecules longer than 200 nucleotides were purified and with the employed standard protocol, all short RNA molecules with length less than 200 nucleotides were removed. These removed small molecules include most of the ncRNA and short mRNA. The quantity and integrity of the purified DNA and RNA was verified using the NanoDrop 1000 Spectrophotometer V3.7 (PeqLab) and the 2100 Bioanalyzer (Agilent), which uses an on-chip electrophoresis. For the assessment of gene expression of candidate adipokines with PCR, 2µg

RNA were reverse transcribed to cDNA using the High Capacity cDNA Reverse Transcription Kit (Applied Biosystems).

Library preparation & multiplexing

The extracted RNA was prepared for sequencing using the TruSeq RNA and DNA Sample Preparation Kit v2 (Illumina). First, it was polyA-selected to purify and enrich for mRNA, fragmented into small pieces and primed (with random hexamers) for cDNA synthesis. The cDNA products were then enriched with PCR and ligated to adapters to multiplex samples. In the ligation, single indexes were used. Finally, the cDNA libraries were created, validated, normalized (so that they had equal volume) and pooled. The resulting pooled single-indexed paired-end libraries of different samples were then applied to the flow cells (containing 8 lanes) on cBot (Illumina), so that multiple samples could be sequenced together.

Sequencing and demultiplexing

The multiplexed probes were sequenced on the Illumina HiSeq 2000 platform in 201 sequencing cycles. Of the 200 samples with total RNA extracted from the SAT biopsies, 198 samples were sequenced with 6 samples per lane, yielding on average 64,095,856 raw reads (SD=7,518,970), with minimum 43,373,110 and maximum 85,591,020 raw reads per probe. Two samples were sequenced each on one lane as a sensitivity check and to assess how many more genes can be detected with a greater sequencing depth. After the alignment and QC filtering (see description below), approx. 50 million single reads (i.e., about 25 million paired reads) were available at high quality per sample, and about 330 million single reads of the two deeply sequenced samples.

The raw data containing the sequences of quality-scored base calls was saved in .bcl files. Next, the CASAVA (Illumina) software was used for converting the .bcl files to .fastq files. In the same step, the multiplexed samples were demultiplexed and the raw reads of each sample are extracted and saved.

Quality control of the raw sequencing reads

For an assessment of the sequencing quality, the Q-score was used as quality scoring method, which is a prediction of the probability of an incorrect base call. All probes had a high percentage of bases with high quality (Q>30), on average 88.0% (SD=1.9%). The mean quality score of bases in a probe was 32.5 (SD=0.5).

More quality checks were performed by investigating the sequence quality, GC content, sequence base content, the presence of adapters and duplicated reads using the FASTQC tool[2]. The sequence quality was high for all probes, across all base pair positions of all reads. The GC distribution across all reads was close to the expected theoretical distribution for all probes, with only minor deviations from some probes which didn't indicate systematic biases or deviations. There was no indication for any problems with adapters. Due to the PCR-steps involved in the cluster generation in RNA-Seq, duplicate sequences were naturally observed, around 50% for all probes. There were no specific sequences that were reported to be systematically duplicated, hence there didn't seem to be any systemic problems.

As a summary, the quality control checks of the raw reads showed a consistently high read quality without indication for systematic sequence biases, presence of adapter sequences, or duplication levels beyond what can be expected from RNA-Seq. Hence, the fastq files were parsed to the alignment stage with TopHat2 without trimming or deletion of reads.

Read alignment

The reads were aligned to the human reference genome GRCh38 (Homo_sapiens.GRCh38.78) using TopHat2 (version 2.0.12) with Bowtie2 (version 2.0.6.0) and samtools (version 0.1.18.0), which maps the RNA reads in the presence of insertions, deletions and gene fusions. In the alignment, all reads were used without trimming or discarding reads with low-quality calls.

Quality control of the aligned reads

Low-quality reads, reads with multiple alignments, and not properly aligned pairs were filtered. In more detail, on average, 90.3% (SD=1.2%) of all reads could be aligned. Furthermore, on average, 85.7% (SD=1.9%) or all raw reads could be aligned in pairs and on average 91.8% (SD=1.3%) of the mapped reads had a high mapping quality. Hence, in addition to the high sequencing quality, the alignments had high quality.

Read counts as raw measures of gene expression levels

The aligned reads were first sorted using samtools and then htseq-count was used to obtain counts as a raw measure of the gene expression levels. Counts were obtained for genes with respect to Ensembl gene identifiers. Default settings were used to discard aligned reads with mapping quality smaller than 10. Since all mapped reads had either mapping quality higher than 30 or lower than 10, only high-quality-mapped reads were used to obtain counts. Reads overlapping multiple genes were not counted for any gene (based on the default mode "union"). The median absolute number of counted reads was 23,850,000. The percentage of mapped and counted reads relative to the raw reads was on average 56.7% (SD=3.0%).

Quality control and normalization of read counts

The Ensembl database[3,4] lists in total 64,253 genes for the reference genome GRCh38.78 (obtained from http://dec2014.archive.ensembl.org/index.html). Based on the obtained raw gene expression levels, 48,126 genes were expressed in at least one probe. Interestingly, only 107 of the 48,126 genes (≈0.2%) were uniquely observed in the 2 deeply sequenced probes which yielded 6 times as many reads. On average, the expression of 27,690 genes was observed per sample (SD=1,274, min=25,430, max=33,280), 19,460 genes had at least 5 counts per sample (SD=1,042, min=17,600, max=25,910), 17,500 genes had at least 10 counts per sample (SD=940, min=15,890, max=23,580), and 13,630 genes had at least 50 counts per sample (SD=696, min=12,420,

max=18,470). When investigating the changes when probes were sequenced at the depth of 1 sample per lane, 3 samples per lane, and 6 samples per lane, as can be expected, the number of genes without observed counts decreases, and the average count per gene as well the maximum number of counts per gene increases, but the shape of the average observed counts per gene and the overall pattern don't change with sequencing depth. For further details regarding the comparison of different sequencing depths, the relative frequency of gene classes that are detected with the different sequencing depths was investigated. As could be expected, this percentage is always higher for the deeper sequenced probe, but again, the differences are not substantial especially for protein coding genes.

In the analysis of gene expression measures from RNA-Seq, the observed counts are often dependent on the gene length (the longer the gene, the higher the counts), which can affect downstream analyses.[5] To normalize gene expression measures we computed TPM[6] (transcripts per million: counts per gene length adjusted for total counts per gene lengths in million) as within-sample normalizations to correct for gene length and library size (i.e., total number of reads). TPM-normalized counts were computed for the 48,019 genes which had non-zero counts in at least 1 sample. For this, the gene lengths were obtained from the Ensembl database through biomaRt[7] at dec2014.archive.ensembl.org (accessing GRCh38.78). In addition to the within-sample normalization, the TMM[8] method was used for a between sample normalization to account for potential sequencing biases. TMM computes the trimmed mean of M-values between each pair of samples and thereby normalizes for RNA composition using scaling factors for the total number of reads (i.e., library sizes), which minimizes the log-fold changes between samples. The effective library size is computed and used instead of the observed library size.

We checked the GC content of a gene, which can bias downstream analyses.[5,9] The results indicated that the mean gene expression (i.e., TMM-normalized TPM value over all samples) was not

associated with GC content of all genes. As a result, the gene expression measures were not further normalized for GC content in order not to remove true biological variance and the TMM-normalized TPM values were used as primary measure of gene expression. All genes with observed counts for at least one individual were passed on to the following stages of the data processing.

As further validity check of the obtained gene expression measures that are analyzed in the following, correlations between RNA-Seq and qPCR gene expression estimates were computed for 6 candidate genes that were investigated in Konigorski et al. (2019)[1]. The (Pearson) correlations were r=0.36 for FABP4, r=0.56 for leptin receptor, r=0.58 for adiponectin, and r=0.85-0.87 for leptin, interleukin-6, and resistin, in line with previous reports in the literature.[10,11,12]

Transformations of normalized read counts

Next, the distribution of the gene expression measures was investigated. Transformations (such as a log-transformation) lead to problems for very lowly expressed genes, where almost everyone has an observed count of 0. Hence, the following analyses and transformations were restricted to those 30,917 genes, where at least 25% of the people (i.e., at least 50 probes) had non-zero observed counts. Most lowly expressed genes had a very skewed distribution: of the 30,917 genes, 15,569 had skewness greater than 1, and 5498 had skewness greater than 2.

The skewed genes (i.e., with skewness greater than 1) were transformed using the Yeo-Johnson transformation (which is the Box-Cox transformation of U + 1 for nonnegative values, and of |U| + 1 with parameter 2-λ for U negative) using the yjPower() function in the car R package[13], where the parameter λ for the transformation is determined in a first step using the powerTransform() function. This approach seemed to be successful in removing the skewness for all genes. Still, 7,953 genes didn't seem to be normally distributed according to the Kolmogorov-Smirnoff test with a p-value smaller than 0.001, for example, due to bimodal or other forms of distributions. However, for those genes, there wasn't a clear indication which transformation could yield normally

distributed measures. Hence, the Yeo-Johnson transformed gene expression measures were used as final measure for the analysis. To incorporate that the normality of some genes is questionable, robust analyses incorporating multiple genes (e.g. the GO term enrichment analysis) were performed, and top associated genes with highest significance in the main analyses were inspected manually.

Assessment of genetic variation

In order to investigate genetic variants, SNVs (in coding regions) were called from the RNA-Seq reads using the mpileup tool of bcftools version 1.9[14,15] and further quality-controlled and trimmed. In total, 2,671,949 SNVs and indels were called. After quality-checks and filtering (trimming unseen alternative alleles, keeping only non-monomorphic biallelic SNVs with average depth >5 and average genotype quality >15), 156,776 biallelic SNVs were retained. Further filtering by Hardy-Weinberg equilibrium HWE (p-value $<10^{-7}$) and minor allele frequency (MAF)<0.01 left 138,244 autosomal biallelic non-monomorphic quality-controlled SNVs. Of them, 97,376 SNVs were used for imputation with Beagle 5.0[16] (Browning et al. 2018) with the 1000 Genomes Project phase 3 reference panel[17]. This yielded 4,796,118 autosomal biallelic non-monomorphic quality-controlled SNVs, which contained 71,093 singletons with a MAF of 0.0025 and 41,447 doubletons with MAF=0.005. Furthermore, 500,430 SNVs had MAF between 0.005 and 0.01, 1,435,935 SNVs had MAF between 0.01 and 0.05, and 2,747,213 SNVs had MAF greater than 0.05. For the complete-case analysis in the sample of n=160, 4,776,233 non-monomorphic SNVs were available.

GO-term enrichment analysis

For the GO-term enrichment analysis, the topGO R package[18] was used with the biological processes (”BP”) sub-ontology, pruning GO terms with less than 10 annotated genes before the enrichment analysis, and computing gene-GO term mappings based on the Ensembl gene identifiers. As enrichment tests, classic Fisher’s exact test and the classic as well as adapted ”elim”

Kolmogorov-Smirnoff (KS) gene-set enrichment were performed. For a summary of the results, the differentially distributed GO terms of these genes (after adjusting for multiple testing of all GO terms) were traced back to the level 1 (i.e. highest order) GO terms and counted for SAT and SAT/TAT, separately.

References

1. Konigorski, S., Janke, J., Drogan, D., Bergmann, M.M., Hierholzer, J., Kaaks, R., Boeing, H., and Pischon, T. (2019). Prediction of circulating adipokine levels based on body fat compartments and adipose tissue gene expression. Obes. Facts *12*, 590–605.
2. Andrews, S. (2010). FastQC: a quality control tool for high throughput sequence data. Available at: http://www.bioinformatics.babraham.ac.uk/projects/fastqc.
3. Aken, B.L., Ayling, S., Barrell, D., Clarke, L., Curwen, V., Fairley, S., Banet, J.F., Billis, K., Girón, C.G., Hourlier, T., et al. (2016). The Ensembl gene annotation system. Database: The Journal of Biological Databases and Curation: baw093.
4. Hubbard, T., Barker, D., Birney, E., Cameron, G., Chen, Y., Clark, L., Cox, T., Cuff, J., Curwen, V., Down, T., et al. (2002). The Ensembl genome database project. Nucleic Acids Res. *30*, 38–41.
5. Hansen, K.D., Irizarry, R.A., and Zhijin, W. (2012). Removing technical variability in RNA-Seq data using conditional quantile normalization. Biostatistics *13*, 204–216.
6. Li, B., and Dewey, C.N. (2011). RSEM: accurate transcript quantification from RNA-Seq data with or without a reference genome. BMC Bioinformatics *12*, 323.
7. Durinck, S., Spellman, P.T., Birney, E., and Huber, W. (2009). Mapping identifiers for the integration of genomic datasets with the R/Bioconductor package biomaRt. Nat. Protoc. *4*, 1184–1191.
8. Robinson, M.D., and Oshlack, A. (2010). A scaling normalization method for differential expression analysis of RNA-seq data. Genome Biol. *11*, R25.
9. Risso, D., Schwartz, K., Sherlock, G., Dudoit, S. (2011). GC-content normalization for RNA-Seq data. BMC Bioinformatics *12*, 480.

10. Marioni, J.C., Mason, C.E., Mane, S.M., Stephens, M., and Gilad, Y. (2008). RNA-seq: an assessment of technical reproducibility and comparison with gene expression arrays. Genome Res. *18*, 1509–1517.
11. Wang, Z., Gerstein, M., and Snyder, M. (2009). RNA-Seq: a revolutionary tool for transcriptomics. Nat. Rev. Genet. *10*, 57–63.
12. Lee, S., Seo, C.H., Lim, B., Yang, J.O., Oh, J., Kim, M., Lee, S., Lee, B., Kang, C., and Lee, S. (2011). Accurate quantification of transcriptome from RNA-Seq data by effective length normalization. Nucleic Acids Res. *39*, e9.
13. Fox, J., and Weisberg, S. (2011). An R companion to applied regression. Thousand Oaks CA: Sage.
14. Li H (2011). A statistical framework for SNP calling, mutation discovery, association mapping and population genetical parameter estimation from sequencing data. Bioinformatics *27*, 2987–2993.
15. Li H, Handsaker B, Wysoker A, Fennell T, Ruan J, Homer N, et al. (2009). The Sequence alignment/map (SAM) format and SAMtools. Bioinformatics *25*, 2078–2079.
16. Browning BL, Zhou Y, Browning SR (2018). A one-penny imputed genome from next generation reference panels. Am. J. Hum. Genet. *103*, 338–348.
17. 1000 Genomes Project Consortium, Auton A, Brooks LD, Durbin RM, Garrison EP, Kang HM, et al. (2015). A global reference for human genetic variation. Nature 526, 68–74.
18. Alexa A, Rahnenführer J (2016). topGO: enrichment analysis for Gene Ontology. R package version 2.28.0.

Supplementary Tables

GO terms	SAT	SAT/TAT
Cellular process	186	174
Biological regulation	158	95
Metabolic process	49	99
Immune system process	84	55
Response to stimulus	84	49
Localization	27	46
Biological adhesion	16	14
Cell proliferation	14	12
Cellular component organization or biogenesis	30	9
Developmental process	24	7
Locomotion	8	20
Multi-organism process	13	2
Multicellular organismal process	27	11
Signaling	33	10

Table S1. Results of the GO term enrichment analysis in the obesity study.

Shown are the number of (highest-level parent GO terms of those) GO terms that were under-/overrepresented in the 441 genes associated with SAT (left panel) and 225 genes associated with SAT/TAT (right panel), compared to the full pool of 30,917 genes, using the classical KS test.

SAT			SAT/TAT		
**#**	**GO ID**	**GO term**	**#**	**GO ID**	**GO term**
1	0002376	immune system process	1	0044710	single-organism metabolic process
2	0006955	immune response	2	0044710	small molecule metabolic process
3	0002682	regulation of immune system process	3	0044699	single-organism process
4	0001775	cell activation	4	0044763	single-organism cellular process
5	0045321	leukocyte activation	5	0002376	immune system process
6	0002252	immune effector process	6	0001775	cell activation
7	0002684	positive regulation of immune system process	7	0006955	immune response
8	0016192	vesicle-mediated transport	8	0045321	leukocyte activation
9	0050776	regulation of immune response	9	0055114	oxidation-reduction process
10	0051234	establishment of localization	10	0006082	organic acid metabolic process
11	0046649	lymphocyte activation	11	0002684	positive regulation of immune system process
12	0051179	localization	12	0043436	oxoacid metabolic process
13	0050778	positive regulation of immune response	13	0002682	regulation of immune system process
14	0006810	transport	14	0032787	monocarboxylic acid metabolic process
15	0002263	cell activation involved in immune response	15	1902578	single-organism localization
16	0042110	T cell activation	16	0006954	inflammatory response
17	0002366	leukocyte activation involved in immune response	17	0019752	carboxylic acid metabolic process
18	0019882	antigen processing and presentation	18	0044765	single-organism transport
19	0048002	antigen processing & presentation of peptide antigen	19	0006091	generation of precursor metabolites and energy
20	0050896	response to stimulus	20	0006629	lipid metabolic process

Table S2. Results of the GO term enrichment analysis in the obesity study, of the 441 genes associated with SAT (left panel) and 225 genes associated with SAT/TAT (right panel).

Shown are the top 20 GO terms with smallest p-values based on the classic KS test.

GO ID	GO term	Annotated	Significant	Expected	classicFisher
GO:0046903	secretion	1421	64	33.6	3.10×10^{-7}
GO:0045055	regulated exocytosis	714	40	16.88	3.60×10^{-7}
GO:0006887	exocytosis	823	43	19.46	8.40×10^{-7}
GO:0016192	vesicle-mediated transport	1758	73	41.56	9.10×10^{-7}
GO:0002275	myeloid cell activation involved in immune response	525	32	12.41	9.60×10^{-7}
GO:0008283	cell proliferation	1799	74	42.53	1.10×10^{-6}
GO:1902578	single-organism localization	2956	107	69.89	1.30×10^{-6}
GO:0042127	regulation of cell proliferation	1421	62	33.6	1.50×10^{-6}
GO:0032940	secretion by cell	1308	58	30.92	2.00×10^{-6}
GO:0044699	single-organism process	11716	310	277	3.50×10^{-6}
GO:0002252	immune effector process	1033	48	24.42	5.10×10^{-6}
GO:0009605	response to external stimulus	1842	73	43.55	5.10×10^{-6}
GO:0002274	myeloid leukocyte activation	600	33	14.19	6.00×10^{-6}
GO:0043299	leukocyte degranulation	519	30	12.27	6.20×10^{-6}
GO:0002376	immune system process	2489	91	58.85	7.50×10^{-6}

Table S3. Results of the GO term enrichment analysis for SAT-associated genes in the obesity study.

Shown are the 15 GO terms that were (statistically significantly after multiple testing correction for all 6287 analyzed GO terms) overrepresented in the 441 genes associated with SAT compared to the full pool of 30,917 genes, using Fisher's exact test. Shown are the GO terms with their description, the number of genes that were annotated with the respective term in all 30,917 genes ("Annotated"), the number of genes that were annotated with the respective term in the 441 SAT-associated genes ("Significant"), which is contrasted with the expected number of annotated genes in this pool of 441 genes ("Expected"), and the p-value from Fisher's exact test.

**GO.ID**	**Term**	**Annotated**	**Significant**	**Expected**	**classicFisher**
GO:0016054	organic acid catabolic process	223	22	2.8	6.90×10^{-14}
GO:0046395	carboxylic acid catabolic process	223	22	2.8	6.90×10^{-14}
GO:0043436	oxoacid metabolic process	997	44	12.52	1.20×10^{-13}
GO:0006082	organic acid metabolic process	1013	44	12.72	2.00×10^{-13}
GO:0055114	oxidation-reduction process	946	42	11.88	3.90×10^{-13}
GO:0019752	carboxylic acid metabolic process	890	40	11.17	1.10×10^{-12}
GO:0044281	small molecule metabolic process	1925	61	24.17	2.00×10^{-12}
GO:0044282	small molecule catabolic process	329	23	4.13	2.50×10^{-11}
GO:0044710	single-organism metabolic process	3990	89	50.1	8.10×10^{-10}
GO:0009063	cellular amino acid catabolic process	109	13	1.37	1.00×10^{-9}
GO:0032787	monocarboxylic acid metabolic process	512	26	6.43	1.30×10^{-9}
GO:0072329	monocarboxylic acid catabolic process	111	13	1.39	1.30×10^{-9}
GO:0044712	single-organism catabolic process	890	35	11.17	1.30×10^{-9}
GO:0044283	small molecule biosynthetic process	489	25	6.14	2.40×10^{-9}
GO:1901606	alpha-amino acid catabolic process	94	11	1.18	2.50×10^{-8}
GO:0006631	fatty acid metabolic process	318	18	3.99	1.10×10^{-7}
GO:0009083	branched-chain amino acid catabolic proc...	20	6	0.25	1.20×10^{-7}
GO:0009062	fatty acid catabolic process	91	10	1.14	2.00×10^{-7}
GO:0006635	fatty acid beta-oxidation	70	9	0.88	2.20×10^{-7}
GO:0009081	branched-chain amino acid metabolic proc...	23	6	0.29	3.10×10^{-7}
GO:0019395	fatty acid oxidation	95	10	1.19	3.10×10^{-7}
GO:0044242	cellular lipid catabolic process	175	13	2.2	3.20×10^{-7}
GO:0034440	lipid oxidation	97	10	1.22	3.70×10^{-7}
GO:1901605	alpha-amino acid metabolic process	211	14	2.65	4.40×10^{-7}
GO:0015980	energy derivation by oxidation of organi...	253	15	3.18	7.20×10^{-7}
GO:0035384	thioester biosynthetic process	65	8	0.82	1.50×10^{-6}
GO:0071616	acyl-CoA biosynthetic process	65	8	0.82	1.50×10^{-6}
GO:0016042	lipid catabolic process	269	15	3.38	1.50×10^{-6}
GO:1901565	organonitrogen compound catabolic proces...	343	17	4.31	1.60×10^{-6}
GO:0016053	organic acid biosynthetic process	279	15	3.5	2.40×10^{-6}
GO:0046394	carboxylic acid biosynthetic process	279	15	3.5	2.40×10^{-6}
GO:0044711	single-organism biosynthetic process	1373	38	17.24	2.50×10^{-6}
GO:0009108	coenzyme biosynthetic process	151	11	1.9	3.20×10^{-6}
GO:0051186	cofactor metabolic process	378	17	4.75	5.70×10^{-6}
GO:0050900	leukocyte migration	380	17	4.77	6.20×10^{-6}
GO:0006091	generation of precursor metabolites and ...	343	16	4.31	6.90×10^{-6}

Table S4. Results of the GO term enrichment analysis for SAT/TAT-associated genes in the obesity study.

Shown are the 36 GO terms that were (statistically significantly after multiple testing correction for all 6287 analyzed GO terms) overrepresented in the 225 genes associated with SAT/TAT compared to the full pool of 30,917 genes, using Fisher's exact test. Shown are the GO terms with their description, the number of genes that were annotated with the respective term in all 30,917 genes ("Annotated"), the number of genes that were annotated with the respective term in the SAT/TAT-associated genes ("Significant"), which is contrasted with the expected number of annotated genes in this pool of 225 genes ("Expected"), and the p-value from Fisher's exact test.

see file “TableS5.xlsx”

Table S5. Results and annotations for the 430 autosomal genes associated with SAT mass.

Shown are the Ensembl ID of the gene (“Ensembl”), the gene symbol (“Gene”), the genomic position in form of the chromosome number (“Chr”) as well as the start (“Gene_start”) and end (“Gene_end”) position in base pairs of the gene; whether the gene is known to be associated with obesity (based on the NCBI gene and GWAS Catalog databases), the corresponding protein encoded by the gene if it is known (from UniProt); the estimates of the effect size (“C-JAMP_beta”; estimate of beta_j in the marginal model of C-JAMP in equation (1) in the main text), its standard error estimates (“C-JAMP_SE”) and p-value (“C-JAMP_pvalue”) from the C-JAMP association analysis of SAT and SAT/TAT conditional on the gene expression and covariates; the results from the Mendelian randomization (MR) analysis in form of the number of SNVs in the gene that were incorporated in the MR analysis (“MR_no_var”), the explained variance of the gene expression based on a multiple linear regression model containing these SNVs (“MR_R2”), and the p-value from the inverse-variance weighted MR method (“MR_pvalue”); as well as the results of the replication analysis in form of the number of SNVs in the gene that were incorporated in the MR analysis (“no_var”), the explained variance of the gene expression based on a multiple linear regression model containing these SNVs (“R2”), and the p-value from the linear regression analysis (“pvalue”).

see file “TableS6.xlsx”

Table S6. Results and annotations for the 215 autosomal genes associated with SAT/TAT.

Shown are the Ensembl ID of the gene (“Ensembl”), the gene symbol (“Gene”), the genomic position in form of the chromosome number (“Chr”) as well as the start (“Gene_start”) and end (“Gene_end”) position in base pairs of the gene; whether the gene is known to be associated with obesity (based on the NCBI gene and GWAS Catalog databases), the corresponding protein encoded by the gene if it is known (from UniProt); the estimates of the effect size (“C-JAMP_beta”; estimate of beta_j in the marginal model of C-JAMP in equation (2) in the main text), its standard error estimates (“C-JAMP_SE”) and p-value (“C-JAMP_pvalue”) from the C-JAMP association analysis of SAT and SAT/TAT conditional on the gene expression and covariates; the results from the Mendelian randomization (MR) analysis in form of the number of SNVs in the gene that were incorporated in the MR analysis (“MR_no_var”), the explained variance of the gene expression based on a multiple linear regression model containing these SNVs (“MR_R2”), and the p-value from the inverse-variance weighted MR method (“MR_pvalue”); as well as the results of the replication analysis in form of the number of SNVs in the gene that were incorporated in the MR analysis (“no_var”), the explained variance of the gene expression based on a multiple linear regression model containing these SNVs (“R2”), and the p-value from the linear regression analysis (“pvalue”).

Characteristics	Women	Men
**Sample size**	2571	2333
**Age,** years	55.0 (7.4)	56.7 (7.5)
**Smoking,** %		
never	62.9	54.6
previous	31.7	37.7
current	5.4	7.8
**Vocational training,** %		
College/university	39.2	45.8
A/AS levels	15.1	12.9
0 levels/GCSE	23.8	17.6
CSE	4.8	4.7
NVQ/HND/HNC	3.5	7.6
Other	13.5	11.3
**BMI,** kg/m^2	26.3 (4.6)	27.2 (3.8)
**aSAT,** kg*	7.5 (3.1)	5.5 (2.0)
**VAT,** kg*	2.4 (1.5)	4.7 (2.3)

Table S7. Sex-stratified characteristics of the study population from the UK Biobank (n=4904).

Values are relative frequencies, mean and SD, or *median and median absolute deviation. aSAT, abdominal subcutaneous adipose tissue; BMI, body mass index; CSE, Certificate of Secondary Education; GCSE, General Certificate of Secondary Education; HNC, Higher National Certificate; HND, Higher National Diploma; NVQ, National Vocational Qualifications; VAT, visceral adipose tissue.

Supplementary Figures

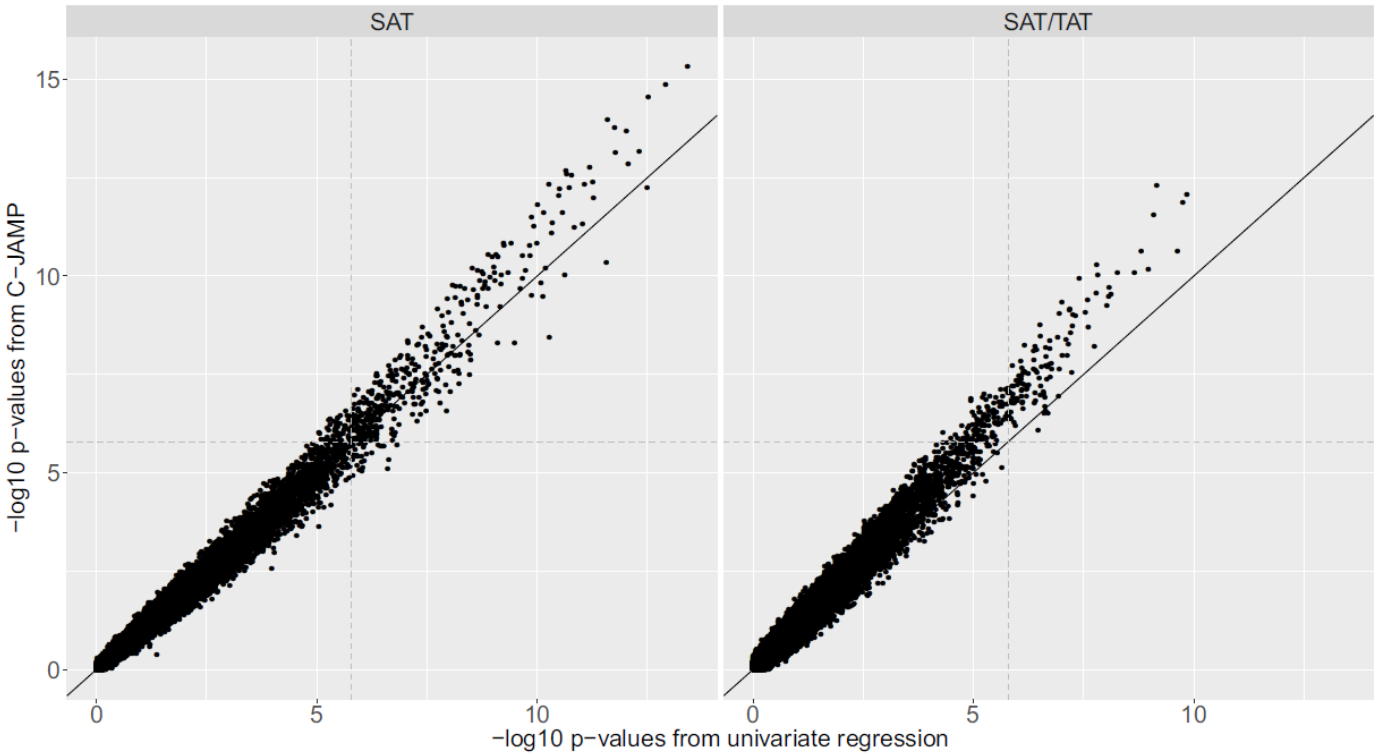

Figure S1. Scatterplots of p-values from the copula analysis C-JAMP versus linear regression from the transcriptomic association analysis with obesity traits.

P-values are on a −log10 scale from C-JAMP models of SAT and SAT/TAT conditional on gene expression and covariates with grey dashed lines at the Bonferroni-adjusted significance threshold $\alpha = 3.2 \times 10^{-5}$.
